# Assessment of Soil Heavy Metal Pollution and Health Risks in Different Functional Areas on the Northern Slope of the Eastern Tianshan Mountains in Xinjiang, NW China

**DOI:** 10.3390/ijerph20064843

**Published:** 2023-03-09

**Authors:** Halidan Asaiduli, Abdugheni Abliz, Abudukeyimu Abulizi, Xiaoli Sun, Panqing Ye

**Affiliations:** 1 College of Geography and Remote Sensing Science, Xinjiang University, Urumqi 830046, China; halida0317@126.com (H.A.);; 2 Xinjiang Key Laboratory of Oasis Ecology, Xinjiang University, Urumqi 830046, China; 3 Key Laboratory of Smart City and Environment Modelling of Higher Education Institute, Xinjiang University, Urumqi 830046, China; 4 Ecological Post-Doctoral Research Station of Xinjiang University, Urumqi 830046, China

**Keywords:** different functional areas, soil heavy metal, spatial distribution, pollution assessment, source analysis

## Abstract

In typical semi-arid areas, the timely and effective monitoring and evaluation of soil heavy metal pollution are of critical importance to prevent soil deterioration and achieve the sustainable use of soil resources. To further understand the degree of soil heavy metal pollution in different functional areas, we studied the soil heavy metal pollution on the northern slope of the eastern Tianshan Mountains in Xinjiang. We collected 104 surface soil samples from typical commercial (A), industrial (B), and agricultural (C) areas with different land-use patterns. The contents of Zn, Cu, Cr, Pb, As, and Hg in the soil of different functional areas were evaluated using the geo-accumulation index, the single-factor pollution index, and potential ecological risk. The results showed that the contents of Pb, As, and Hg in soils of different functional areas exceeded 4.47, 8.03, and 1.5 times the background values of Xinjiang soil, respectively. The average contents of Zn, Cu, and Cr were lower than the background values of Xinjiang soil. Except for As in different functional areas, the contents of the other elements in the different functional areas reached the level of soil environmental quality standards in China (GB15618-2018). The geo-accumulation index of heavy metals in different functional areas was in the order of Area C > Area A > Area B, indicating that Area C was the most polluted. The results of the single-factor pollution index showed that the pollution levels of Pb, As, and Hg were higher, and the pollution levels of Cr, Cu, and Zn were lower. The results of the potential ecological risk index showed that the northwest of Area A was higher, the southeast of Area B was more polluted, and the central and eastern parts of Area C were more polluted. From the perspective of spatial distribution, the spatial distribution characteristics of Zn and Cr elements in different functional areas are consistent, but the spatial distribution characteristics of Cu, Pb, As, and Hg in different functional areas are quite different. The high values of these four elements are mainly distributed in residential areas, factories, and metal smelters. It is necessary to divide the functional areas based on different land-use patterns, and the prevention of soil single-element and heavy metal pollution in different functional areas is reasonable for land resources and provides a scientific basis for the effective planning of quality assurance.

## 1. Introduction

The emissions of pollutants related to human activities are increasing with the rapid development of urbanization, industrialization, and agricultural intensification. These pollutants enter the soil system and cause soil to be polluted to varying degrees, destroying the structure and function of the soil environment and endangering the ecological environment and human health [1,2,3]. Soil heavy metal pollution is one of the main types of soil pollution [4], and it is a research hotspot in environmental science, geography, and ecology. Previous studies have shown that heavy metal elements enter the soil environment in a variety of ways through the food chain and respiratory system into the human body, causing harm to human health. Zn and Cr are recognized toxic elements; they can change the human nervous system and respiratory function and interfere with the endocrine system [5]. Excessive intake of Cu can cause Cu poisoning, acute hemolysis, and renal dysfunction [6]. Pb has no physiological function in the human body and causes serious harm to human health, including slowing children’s cognitive development and weakening their intelligence [7]. Previous studies have shown that long-term agricultural activities and green management (such as excessive use of fertilizers and pesticides) may lead to the accumulation of As in soil [8,9]. Inorganic arsenic (calcium arsenate, sodium arsenate, and lead arsenate) is widely used in fertilizers to increase crop yield. In recent years, the determination of arsenic species in environmental and industrial sources has become important due to arsenic’s toxic effects on humans [10,11]. As the largest developing country in the world, China’s industry and economy have developed rapidly in the past 40 years. According to the national soil pollution survey bulletin, heavy metal pollution in agricultural soil in southern China is serious [12,13]. However, with the rapid development of industrial and agricultural activities, Hg emissions have also increased significantly [14]. Studies have shown that Hg is very harmful to human health [15]. In the northwest of China, soil heavy metal pollution is very high, and As and Cr are the main heavy metal elements that pose a threat to human health in the region, according to previous studies [16].

A large number of studies have reported that the physicochemical properties of soil could be impacted by different land uses (wetland, grassland, and afforestation) [17]. For example, Imperato [18] and Pavao-Zuckerman [19] found that the values of different heavy metal elements were unevenly distributed in different land-use functional areas. The northern slope of the eastern Tianshan Mountains has the most developed economy. It is the most densely populated area, with the most active industrial and agricultural activities, and the land-use types are abundant. However, due to rapid industrialization in recent years, the region has experienced unpredictable environmental pollution problems [20]. Currently, the development speed of urbanization and the industrial and agricultural levels in northwest China is remarkable, and the problem of soil environmental pollution is a research hotspot. Although some scholars have studied the distribution of heavy metals in the soil of single land-use functional areas such as farmland [21] and watersheds [22], few researchers have focused on the degree of soil heavy metal pollution in different land-use functional areas, especially in the northern slope of the eastern Tianshan Mountains in Xinjiang. Therefore, assessing soil quality under different land uses is extremely important, especially in different functional areas. In this paper, taking the northern slope of the East Tianshan Mountains in Xinjiang, China, as an example, the soil is sampled and analyzed according to different production, living, and commercial land-use characteristics in order to understand the impact of different human activities on soil heavy metal pollution and to provide a scientific basis for environmental planning and protection.

We used different functional areas of the economic belt on the northern slope of the Tianshan Mountains as the research object, collected 104 soil surface layers, and determined the contents of heavy metal elements such as Zn, Cu, Cr, Pb, As, and Hg in the soil. The single-factor pollution index, a geo-accumulation index evaluation, the potential ecological risk index, and the multivariate statistical analysis method were used to evaluate the pollution of soil heavy metals, and the inverse distance weight (IDW) method was used to reveal the spatial distribution characteristics. The specific objectives of this study are (1) to use the geo-accumulation index and the single-factor pollution index to evaluate the degree of soil heavy pollution and the potential ecological risk index to assess the degree of soil heavy metal ecological risk; (2) to use the geostatistical method to quantitative analyze the pollution characteristics and spatial distribution of each area; (3) to use the multivariate statistical method to analyze the main pollution sources. This research provides a scientific basis for the evaluation of soil environmental quality to support the reasonable and effective planning of land resources in different functional areas of the economic belt on the northern slope of the Tianshan Mountains.

## 2. Materials and Methods

### 2.1. Study Area and Soil Sampling Collection

The northern slope economic belt of the Tianshan Mountains (42°45′–46°8′ N, 84°46′–88°58′ E) is located at the northern foot of the Tianshan Mountains in the Xinjiang Uygur Autonomous Region, China. It is located in the southern margin of the Junggar Basin, with an area of 28.25 km^2^, accounting for about 17.20% of the total area of Xinjiang. It has a large diurnal temperature range, and the area belongs to a typical continental arid climate [23,24].

**Figure 1:**
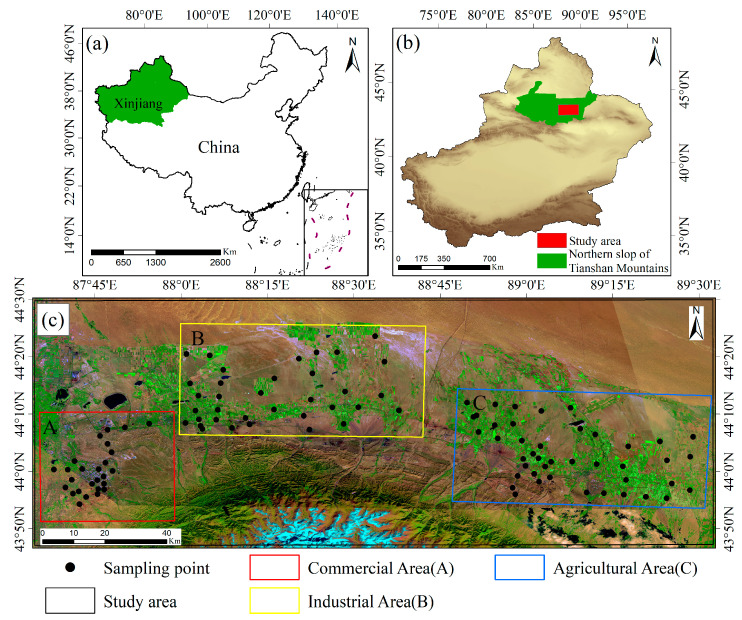
(**a**) Location of the study area on the overview map of China; (**b**) Xinjiang location; (**c**) map of the study area and distribution of sampling points in different functional areas.

To promote regional sustainable development and further reveal the pollution of soil heavy metal elements under different land-use patterns, the functional areas were subdivided according to different plans and the utilization of the urban land. A field survey was conducted on the northern slope of the eastern Tianshan Mountains. According to the different function, living, and commercial characteristics of the study area, samples were collected at the typical dense points for partition sampling analysis. The study area was divided into commercial (A), industrial (B), and agricultural (C) areas, and we used GPS to accurately locate and conduct on-the-spot investigations when collecting soil samples; a total of 104 surface soil samples were collected (Figure 1). The areas where humans are more concentrated and active were classified as Area A, and 26 soil samples were collected near residences, schools, and hospitals. Fukang is an important coal chemical base in Xinjiang. Coal resources are widely distributed, and the reserves are abundant [25]. Therefore, the western part of Fukang, with its dense industrial park, was classified as Area B, and 35 soil surface samples were collected near coal chemical plants and coal power plants. In Fukang, the total area of the coal mining area reached 280 km^2^, and the total design had a mine production scale of 8.49 million tons per year, according to coal and metal smelter owners. The economic belt on the northern slope of the Tianshan Mountains is a famous agricultural base in Xinjiang for growing cash crops such as cotton, wheat, corn, and sunflowers [26]. We collected 62 soil samples near the farmlands in Jimsar and Qitai, and the area was divided as Area C. To accurately reveal the accumulation degree of heavy metals in the soil of different functional areas and ensure that the collected samples were not disturbed by other land-use functional areas, all soil samples were collected from depths of 0–20 cm from the surface soil.

### 2.2. Laboratory Analysis

The collection and analysis of 104 samples from different functional areas (Areas A, B, and C) were completed in 2019. After taking the soil samples back to the laboratory, they were air-dried to remove residues and stones. To prevent heavy metal pollution, metal materials were avoided during the experiment: an agate mortar was used for grinding, and the samples were passed through a 100-mesh (0.25 mm) nylon sieve and stored in polyethylene bottles. The specific chemical test process was as follows: 0.5 g of the sieved soil sample was placed in a tetrafluoroethylene (C_2_F_4_) dry pot. First, 9 mL of concentrated hydrochloric acid (HCI) was added, followed by 3 mL of concentrated nitric acid (HNO_3_). This mixture was heated to allow for evaporation for 20 min; then, 5 mL of hydrofluoric acid (HF) was added and heated for 30 min. Next, 3 mL of perchloric acid (HCIO_4_) was added, and the sample was heated until it was nearly dry. Finally, 1:10 diluted nitric acid was added and evaporated for 15 min. After cooling, the treated sample was diluted with high-purity water to 20 mL [27]. The contents of Zn, Cu, Cr, Pb, As, and Hg were detected. As and Hg were detected by electron fluorescence spectrometry (Tokyo Hitachi High-Tech Company, PF6-2 atomic fluorescence spectrophotometer), and Zn, Cu, Cr, and Pb were detected by flame atomic absorption spectrometry (Tokyo Hitachi High-Tech Company, Hitachi Z-2000 flame atomic absorption spectrophotometer). The same soil sample was measured three times, and the average value was taken. The national standard sample (GSS-4) was used as the quality control sample, and the relative deviation of the determined elements was controlled within ±10%.

### 2.3. Geo-Accumulation Index

The geo-accumulation index (*I_geo_*) was first proposed by the German scholar Muller, so it is also called the Muller index [28]. The *I_geo_* can reveal the pollution caused by natural non-genetic factors [29]. The formula is as follows:

(1) $$I_{geo}=\log_{2}\left(\frac{C_{n}}{1.5\times B_{n}}\right)$$

where *I_geo_* is the geo-accumulation index, *C_n_* represents the measured value, and *B_n_* denotes the background value of the soil. In this study, we use the background value of Xinjiang (mg·kg^−1^) [30]. *I_geo_* is classified as follows: clean (*I_geo_* ≤ 0), light pollution (0 < *I_geo_* ≤ 1), moderate pollution (1 < *I_geo_* ≤ 2), high pollution (2 < *I_geo_* ≤ 3), and very high pollution (*I_geo_* ≥ 3).

### 2.4. Single-Factor Pollution Index

The single-factor pollution index studies the relationship between the measured value of each sampling point and the standard value of the soil. When there are more elements in the soil, it is necessary to comprehensively consider and determine the elements with a greater pollution contribution rate [31]. The formula is as follows:

(2) $$P_{i}=\frac{C_{i}}{B_{n}}$$

where *P_i_* is the single-factor pollution index, *C_i_* is the measured value of the heavy metals *i* in the soil (mg·kg^−1^), and *B_n_* is the geochemical background value of the heavy metals in the local soil; in this study, for *B_n_*, the background value for Xinjiang was used (mg·kg^−1^). The classification standard of the *P_i_* is listed in Table 1.

### 2.5. Potential Ecological Risk Index

To assess the potential impact of pollutants on the ecosystem and evaluate the degree of environmental risk caused by soil heavy metals, the Swedish scientist Hakanson proposed the potential ecological risk index (*PRI*) in 1980 [32,33,34]. The *PRI* can be calculated using the following equations:

(3) $$PRI=\overset{n}{\underset{i}{\sum }}E_{r}^{i}$$

(4) $$E_{r}^{i}=T_{n}^{i}\times C_{r}^{i}$$

(5) $$C_{r}^{i}=\frac{C_{i}}{C_{n}^{i}}$$

where *PRI* is the sum of the potential ecological risk index, $E_{r}^{i}\;$ is the potential ecological risk coefficient of a certain element, $T_{n}^{i}$ is the toxic response coefficient, $C_{r}^{i}$ is the pollution coefficient of the element, $C_{i}$ is the measured value of the element, and $C_{n}^{i}$ is the background value of the element. The classification standard of the PRI is listed in Table 2.

## 3. Results

### 3.1. Statistical Analysis of Heavy Metal Content in Soil

The statistical results of the heavy metal concentrations on the northern slope of the Tianshan Mountains in different functional areas of Zn, Cu, Cr, Pb, As, and Hg are given in Table 3. The average concentrations of the six heavy metal elements in different functional areas are as follows: in Area A, Zn (56.97), Cu (23.27), Cr (33.33), Pb (107.87), As (106.91), and Hg (0.03); in Area B, Zn (58.00), Cu (30.82), Cr (39.56), Pb (70.98), As (89.93), and Hg (0.03); in Area C, Zn (55.80), Cu (28.34), Cr (37.80), Pb (81.19), As (73.09), and Hg (0.03). The average contents of the six elements in the different functional areas were in the following order: Area A (65.67) > Area B (57.86) > Area C (55.24). It can be seen that the pollution of Area A is the most serious, and Pb, As contributed the most to the pollution of the soil environment in the functional area. In the A and C areas, the content of the heavy metal elements was Pb > As > Zn > Cr > Cu > Hg, and Pb pollution was the most serious. In Area C, the contents of the heavy metal elements were as follows: As > Pb > Zn > Cr > Cu > Hg, with As pollution being the most serious; the content of Hg in the different functional areas was similar. The contents of Zn and Cr were lower than the soil background values of Xinjiang. The contents of Cu in Areas A and C were lower than in the soil background values of Xinjiang, but in Area B, it was higher. In addition, based on the soil environmental quality standards of China (GB15618-2018) [35], except for As in different functional areas, the average contents of the other heavy metals in different functional areas reached the standard level. Overall, Pb, As, and Hg contamination in Area A were 5.56, 9.55, and 1.5 times, in Area B 3.66, 8.08, and 1.5 times, and in Area C 4.19, 6.53, and 1.5 times higher than the soil background values of Xinjiang, indicating that the three elements are seriously enriched in the study area. In general, the single heavy metal element pollution in the study area is more serious.

The coefficient of variation (CV) can reflect the degree of the dispersion of the data variables in the spatial distribution and help to evaluate the degree of influence of external factors on variables according to the value [36,37]. If the CV value is 36%, there is strong variation. It can be seen from Table 3 that the Cu elements in Area B and the Cr, As, and Hg elements in Area C are strongly variable, indicating that these elements are more likely to be affected by human activities in the study area.

### 3.2. Assessment of Soil Heavy Metal Pollution in Different Functional Areas

#### 3.2.1. Result of the *I_geo_*

**Figure 2:**
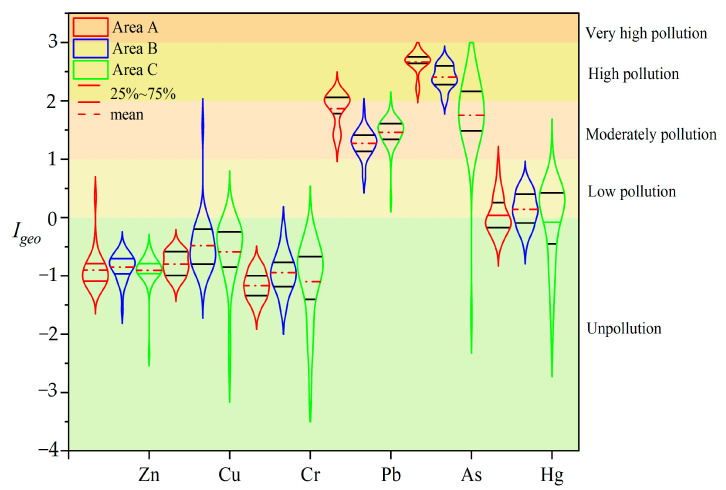
The contamination of heavy metals indicated by the geo-accumulation index (*I_geo_*) in the different functional areas.

The results of the geo-accumulation index (*I_geo_*) of heavy metals in the soil in different functional areas (Figure 2) showed that the geo-accumulation index of Zn, Cu, and Cr was lower than 1, which was at the level of non-pollution to mild pollution, indicating that the pollution of these three elements in each functional area was relatively light. The geo-accumulation index of Pb, As, and Hg in each functional area was less than 3, showing a moderate-to-severe pollution level. The trend of the *I_geo_* of different heavy metal elements in each functional area was Area A (0.50) > Area B (0.43) > Area C (0.41). The pollution of Zn, Cu, and Cr is light and unpolluted, and the pollution of Pb, As and Hg is serious, showing a heavy to extremely heavy pollution level. Therefore, the study area needs to implement a Pb, As, and Hg remediation-based heavy metal pollution control program; however, the remaining heavy metal pollution cannot be ignored. The mean value of heavy metal *I_geo_* in the soil was negative, indicating that the whole site is pollution-free. The detected concentrations of Zn, Cu, Cr, Pb, As, and Hg in some samples exceeded the background values, and the average *I_geo_* in the surface layer was higher than that in the lower layer, which is consistent with the rule that all the sampling sites were unpolluted with the heavy metals Zn, Cu, and Cr. Pb, As, and Hg had different proportions of pollution at the sites, mainly slight pollution.

#### 3.2.2. Result of the *P_i_*

**Figure 3:**
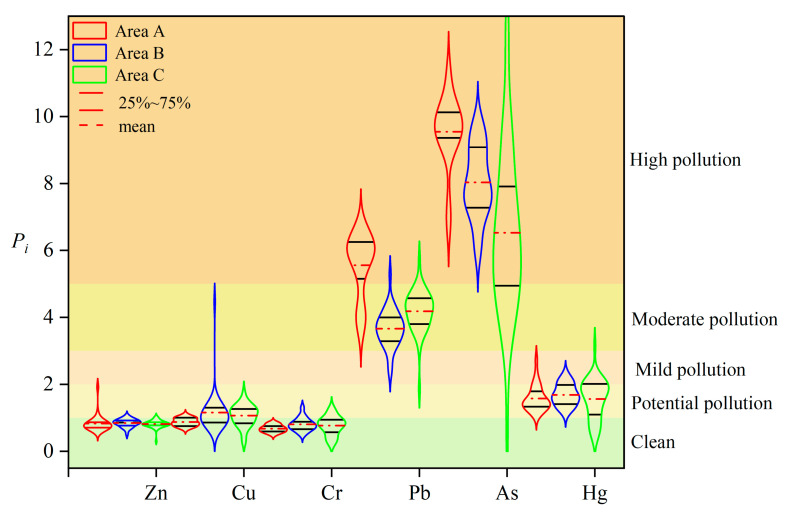
The contamination of heavy metals indicated by the single-factor pollution index (*P_i_*) in the different functional areas.

According to the results of the heavy metal single-factor pollution index (Figure 3), the pollution index of six heavy metals in Areas B and C is As > Pb > Cu > Hg > Cr > Zn, while in Area A, it is As > Pb > Hg > Zn > Cu > Cr, indicating that different functional areas have different contribution rates to element enrichment and industrial activities have a greater impact on the accumulation of Hg, Zn, and Cu. The single-factor pollution indices of Zn, Cu, and Cr were in the range of 0–5, and the pollution level was clean to moderate pollution. The Zn and Cr contents in each functional area were at a clean level; Pb and As were at a heavy pollution level. In general, Pb, As, and Hg pollution in the study area is serious and needs to be resolved.

#### 3.2.3. Result of the Potential Risk Index

**Figure 4:**
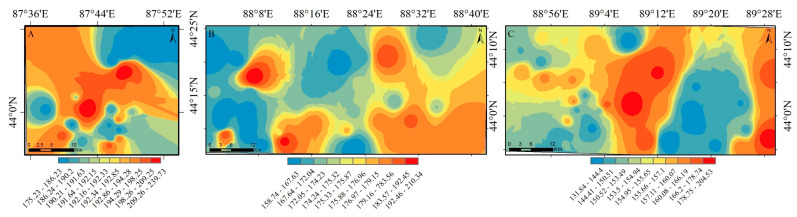
Spatial distribution of heavy metals indicated by the potential risk index (*PRI*) in the areas (**A**–**C**).

The frequency distribution is used to describe the proportion of different levels of potential ecological risk points in the total sample points [38]. The distribution percentages of the potential ecological risk coefficients (*Eri*) of single heavy metals in soil are shown. Each element has been divided into risk levels, and the detailed results are shown in Figure 4. The order of the single-factor potential ecological risk index of six heavy metal elements in the surface soil of the study area is Cd > Pb > Cu > Hg > Cr > Zn. The average value of the potential ecological risk index of Cd far exceeded the maximum value of the risk level, and there was a strong potential ecological risk. The potential ecological risk index showed that Pb was greater than 80, belonging to a strong ecological hazard; the potential ecological risk index of Cu, Zn, and Cr is 600, indicating that the soil in the study area has been seriously polluted by heavy metals and that there is a high ecological risk. Figure 4 indicates that the extremely high risk mainly exists in the northwest and southeast of the smelter and the areas with more human activities. The risk of farmlands away from the factory and living areas is relatively low, but there is still a medium risk. This is similar to the spatial distribution characteristics of the heavy metal content in the surface soil in the study area. The northwest of Area A had a higher risk, the southeast of Area B was more polluted, and the central and eastern parts of Area C were more polluted.

### 3.3. Spatial Distribution of Heavy Metals in Different Functional Areas

**Figure 5:**
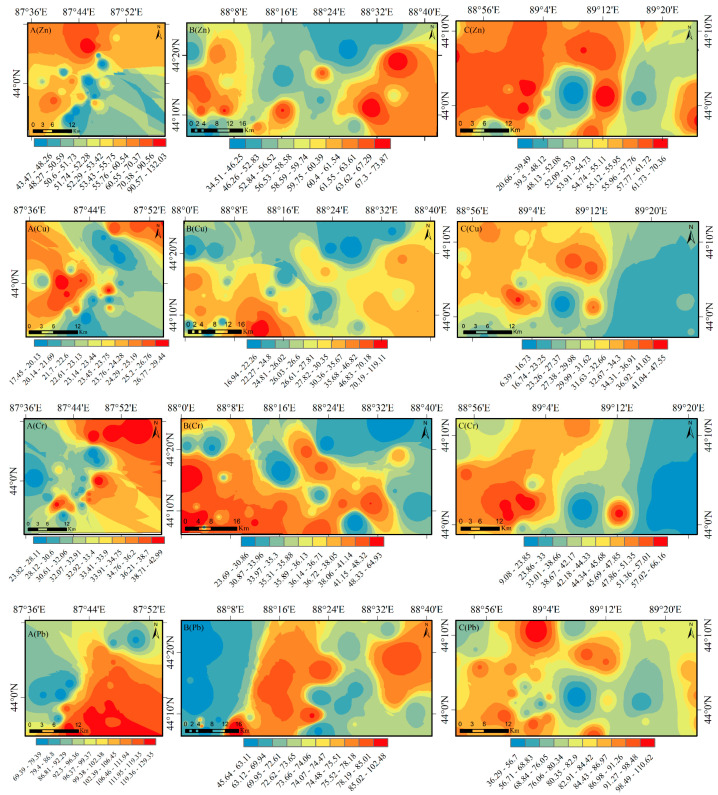

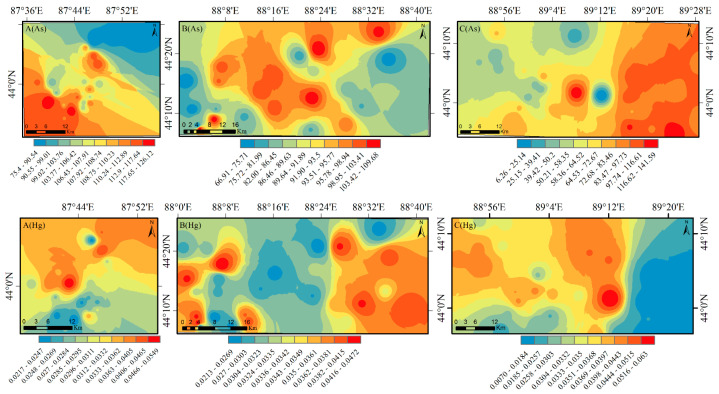
Spatial distribution of heavy metal contents on the northern slope of the Tianshan Mountains in different functional areas (**A**–**C**).

Based on the principle of similar similarity, the inverse distance weight (IDW) method assigns a larger weighted value to a value closer to the interpolation point through weight distribution [39]. In this paper, the spatial distribution patterns of six heavy metal elements in different functional areas are drawn by the IDW method, as shown in Figure 5. From the perspective of spatial distribution, the spatial distribution characteristics of Zn and Cr elements in different functional areas are consistent, but the spatial distribution characteristics of Cu, Pb, As, and Hg in different functional areas are quite different. The high values of these four elements are mainly distributed in residential areas, factories, and metal smelters. Zn, Pb, and As in different functional areas are distributed in sheets, and Cu and Cr are distributed in patches. The high values of the five elements are distributed in the southeast near the metal smelters and coal mines, and the low values appear in the unmanned area, indicating that mining activities have a greater impact on the accumulation of the five elements. The high values of Zn, Cu, Cr, and Pb appear in the western part of the farmland, indicating that agricultural activities have an impact on the accumulation of these four heavy metals. The high values of Pb and As are distributed near the power plants and factories in the east, indicating that wastewater and waste treatment have a greater impact on the accumulation of Pb and As in the production process.

### 3.4. Source Identification of Heavy Metals in Different Functional Areas

#### 3.4.1. Pearson Correlation of Heavy Metals

The correlation coefficient of heavy metal elements in soil can reveal whether the pollutants have the same pollution source. Table 4 shows that the correlation coefficients between Pb-As, Zn-Cr, and Cu-Hg in the study areas are high, reaching a significant positive correlation. The correlation coefficients of Cr-As, Cu-Pb, Cu-As, Pb-Hg, and As-Hg elements in Area A are −0.53, −0.49, −0.49, −0.46 and −0.44, a significant negative correlation, and the correlation of other elements is not strong enough. In Area B, the correlation between Zn-Cr, Zn-Hg, Cu-Cr, Zn-Cu, and Cu-Hg (*p* < 0.05) shows a significant positive correlation and indicates that these three elements have similar pollution sources. The correlation coefficient between As-Zn, As-Cu, As-Cr, and As-Hg Cu-As is a significant negative correlation, indicating that the soil heavy metal pollution sources of these elements may be inconsistent. In Area C, the correlation between Cu-Cr, Cr-Hg, Cu-Hg, Zn-Cu, Zn-Cr, and Zn-Pb exceeds 0.6, reaching 0.87, indicating that the pollution sources of the four heavy metal elements in the C area are consistent. The correlation coefficients of Cr-As, Cu-As, Hg-As, and Zn-As elements in Area C are −0.85, −0.84, 0.80, and −0.62. These elements are significantly negatively correlated, indicating that these soil heavy metal elements in the region may have inconsistent pollution sources.

#### 3.4.2. Principal Component Analysis

Correlation analysis can show the source information between different heavy metal elements and help us speculate about the possible sources [40]. In order to further determine the pollution sources of heavy metals in different functional areas, this study uses SPSS to perform principal component analysis (PCA) on soil heavy metal content (Table 5). The Kaiser–Meyer–Olkin (KMO) test is used to determine the consistency of the data reduction and measures the adequacy of the sample; Bartlett’s characteristic scatter test shows the strength of the correlation between the variables [41]. The practicability of PCA analysis was verified by eigenvalues, percent variance, and total variance before and after the rotation component matrix [42]. In this study, the KMO test results of the A, B, and C areas were 0.65, 0.81, and 0.82 respectively, and the significant Bartlett’s test (significance = 0.00) indicated that data reduction could be performed by PCA. Three principal components were extracted from Area A; the variance contribution rates of PC1, PC2, and PC3 were 40.87%, 20.26%, and 17.36%, respectively, and the cumulative contribution rate reached 78.49%, which could explain the pollution source information. PC1 explains 40.87% of the total variance; the loadings of Cu and Hg were 0.79 and 0.70, respectively, and the factor loading of As and Pb was −0.80 and −0.70, which is consistent with the results of the correlation analysis. There were similar pollution sources among Cu, Pb, and As. PC2 explains 27.94% of the total variance; the loads of Cr and Pb were 0.84 and 0.40, respectively. PC3 explains 20.61% of the total variance, and the load of Zn was 0.95, indicating that the source of Zn element pollution is unique. In general, in Area A, human activities are complex and have a great impact on the accumulation of heavy metals. It is speculated that the pollution in this area is mainly from domestic waste [43].

Two principal components were extracted from Area B. The variance contribution rates of PC1 and PC2 were 48.27% and 17.09%, respectively, and the cumulative contribution rate reached 65.36%, which could summarize most of the information on the heavy metal elements. The loadings of Zn, Cu, Cr, and Hg in PC1 were 0.79, 0.78, 0.74, and 0.63, respectively; the correlation between these four elements is significantly positive, as is the correlation between As and the other elements. The pollution source of Pb in Area B is inconsistent with other elements. In PC2, the load of Pb and Cu was 0.97 and 0.21, and the correlation with other elements is not obvious. According to the spatial distribution characteristics of Pb, the accumulation of Pb is mainly affected by industrial production activities.

Two components were extracted from Area C. The variance contribution rates of PC1 and PC2 were 67.38% and 19.12%, respectively, and the cumulative contribution rate reached 86.51%, which could better explain the pollution source information. In PC1, the loads of Cu, Cr, Hg, Zn, and Pb were 0.92, 0.92, 0.82, 0.76, and 0.48, and the correlation between them is significant, indicating that the five elements have similar pollution sources, that is, mining and metal smelting had a greater impact on the five elements. In PC2, the load of Pb and Zn was 0.80 and 0.48, indicating that Pb and Zn are significantly affected by mining and smelting activities. However, the correlation between Hg and other elements is significantly negative.

**Figure 6:**
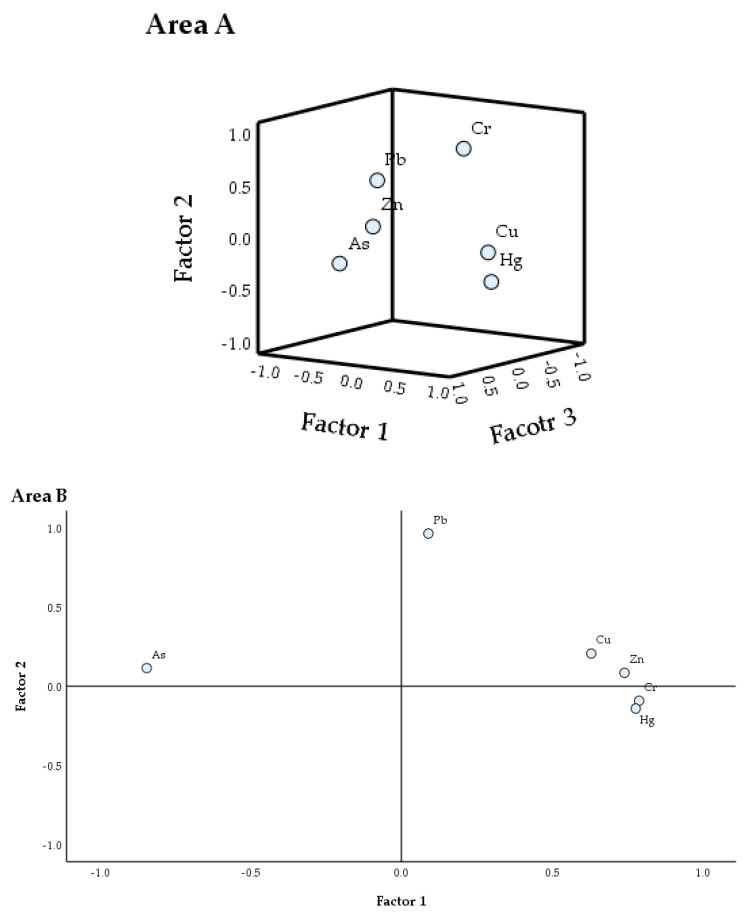

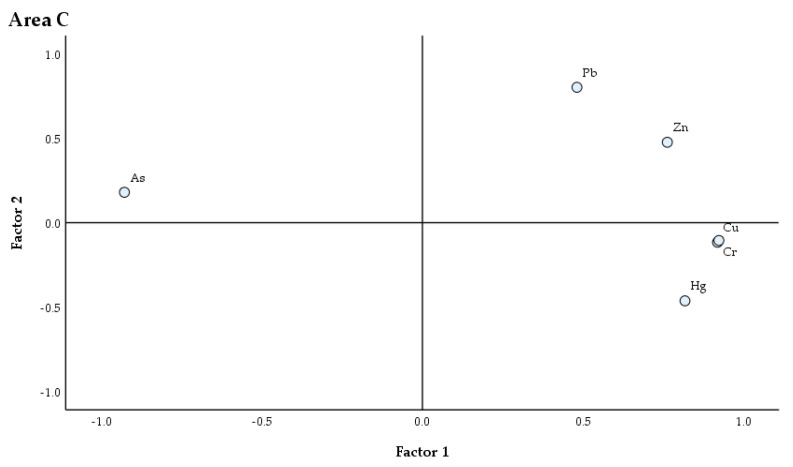
PCA of heavy metal content in surface soil of A, B, C area.

In order to more intuitively reflect the contribution of each evaluation factor in each principal component [44], this study draws a principal component distribution for the content of six heavy metal elements in different functional areas of soil. The variable of the determining factor can be intuitively seen from the load scatter diagram generated by the factor load (Figure 6).

#### 3.4.3. Cluster Analysis

**Figure 7:**
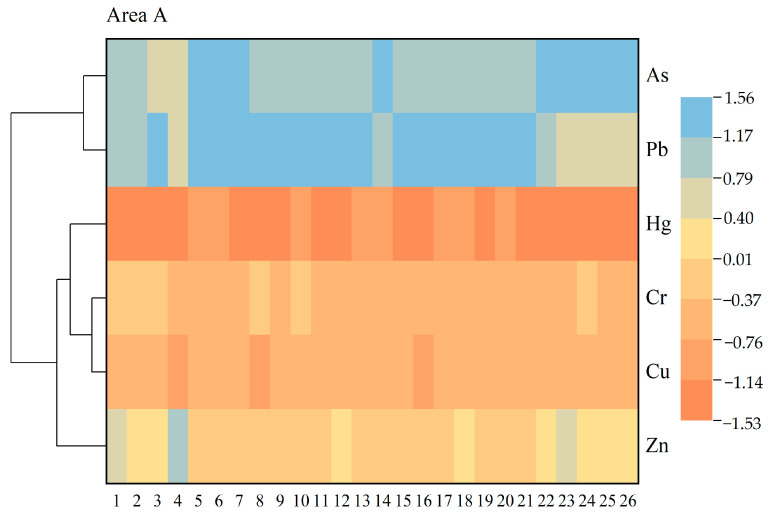

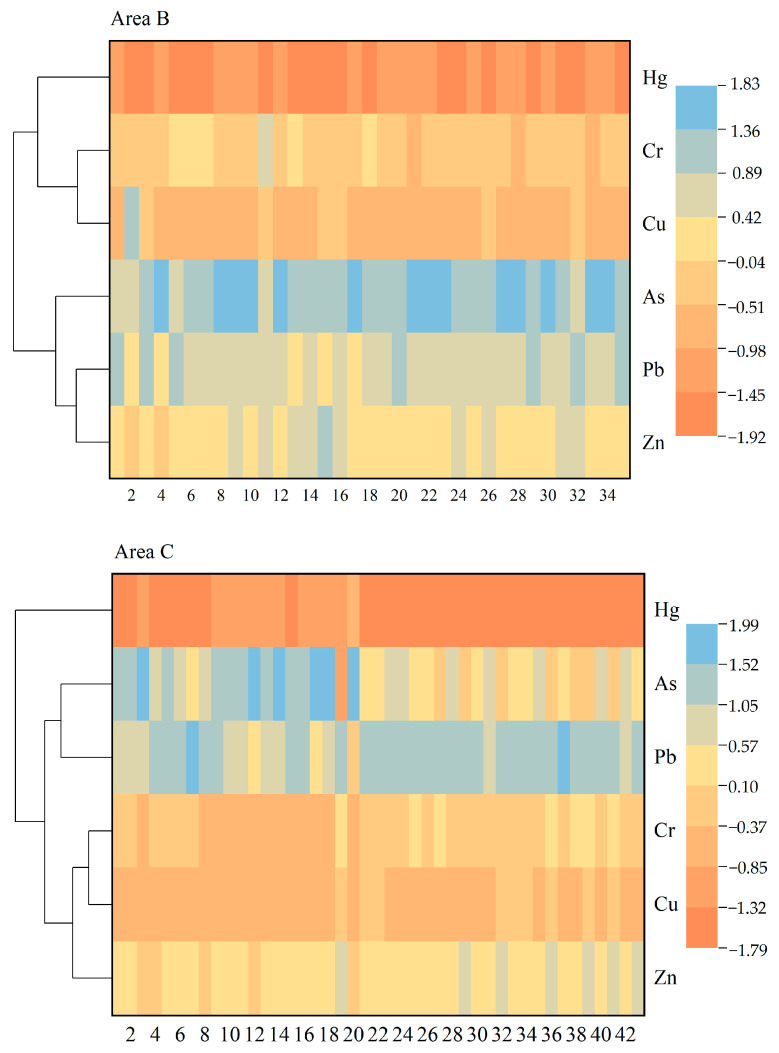
Cluster analysis heat map of heavy metals in different functional areas.

Cluster analysis (CA) is the method that is usually used to analyze the source of heavy metals in more significant detail and to reflect the category of heavy metals. The CA heat map of heavy metals in the soil from different functional areas is shown in Figure 7, and calculating the results revealed the existence of three clusters. Area A includes (1) As, Pb, Cr, and Cu and (2) Hg and Zn; the result is consistent with correlation analysis. Area B includes (1) Cr, Cu, Pb, and Zn, (2) As, and (3) Hg, and according to the correlation analysis result, Cu-Cr is a positive correlation, but the Pb-Zn correlation coefficient is very low. Area C includes (1) As, Pb, Cr, and Cu and (2) Zn and Hg. The CA results are consistent with those derived from PCA.

## 4. Discussion

### 4.1. Soil Heavy Metal Pollution Degree in Relation to Different Functional Areas

Against the background of rapid development of urbanization, industrialization, and agricultural intensification, the change of land resources by human beings has accelerated the diversification of land use on the northern slope economic belt of the Tianshan Mountains in Xinjiang. At present, industrial pollution emissions, mining activities, agricultural production, and more complex human activities pose a serious threat to the soil environment. Our results show that the accumulation degree of soil heavy metal elements in different functional areas varies, and the dominant factors in the different functional areas are also different. From the point of view of the heavy metal content, the average values of Pb, As, and Hg in the soil surface layer of the economic belt on the northern slope of the Tianshan Mountains exceed the soil background value in the Xinjiang soil quality standard, and Zn, Cu, and Cr are lower than the background value of Xinjiang. In addition, except for As in different functional areas, the contents of the other elements in the different functional areas reached the level of soil environmental quality standards in China (GB15618-2018). It shows that industrial activities have a great influence on the accumulation of Cu, Pb, As, and Hg and are obviously enriched in different functional areas. Mamat et al. [45] found that the local enrichment of Cd, Hg, Pb, and Zn elements is caused by human disturbance in the higher geological background in the western region. This is consistent with the analysis of six heavy metal elements (Zn, Cu, Cr, Pb, Hg, and As) in the Zhundong coalfield area by Liu Wei [46] and others. Zn, Cu, and Cr do not show obvious pollution status, and the As pollution greatly exceeds the background value of Xinjiang. From the perspective of spatial distribution and pollution evaluation, Zn, Pb, and As are distributed in sheets and Cu and Cr are distributed in patches. The pollution degree of Areas A and B is more serious, while the pollution degree of Area C is relatively light. The high values of soil heavy metal elements in the heavily polluted areas appear near metal smelters and residential areas with frequent human activities, indicating that the accumulation of heavy metal elements in soil is affected by metal smelting and more complex human activities. This conclusion is consistent with Hu’s research [47].

### 4.2. The Ecological Risk Degree and Source of Soil Heavy Metal Pollution in Different Functional Areas

Soil heavy metals are mainly from natural sources and human disturbance sources. Natural sources mainly include soil texture, atmospheric deposition, weathering decomposition, and litter decomposition. It is considered that the main sources of interference are agricultural chemicals, transportation, industrial activities, and sewage irrigation [48]. The occurrences of soil Zn, Cu, Pb, and Cd in soil are highly correlated with human activities because CaO, SOM, STS, and STP are ranked as the most important factors for their dynamics [49]. The source of Cu pollution mainly comes from the soil parent material, and the source of Zn is more complex, mainly from natural factors; Cr is mainly affected by coal dust and human factors during coal mining [50]. Hu et al. [51] concluded that transportation has a greater impact on Pb, which is consistent with the conclusion of this paper. The accumulation of Cu is related to construction land, and As and Cu are mainly affected by industrial production activities. According to multivariate statistical analysis and spatial distribution characteristics, we conclude that Zn and Cr are mainly from human daily activities, Cu is mainly from industrial sources, Pb is from transportation, and As accumulation is mainly from the application of pesticides and fertilizers.

## 5. Conclusions

In this study, address statistics and multivariate statistical analysis methods were used to evaluate the pollution degree and ecological risk degree of heavy metal elements in the surface soil of different functional areas on the northern slope of the Tianshan Mountains and to quantitatively analyze the sources of pollution. The study found that the degree of heavy metal pollution, ecological risk, and spatial distribution characteristics of soil in different functional areas dominated by land-use patterns are quite different. It is necessary to implement zoning and then carry out pollution source distribution and single-element pollution control measures. The analysis of *I_geo_* and *PRI* results showed that the accumulation of heavy metals was highest in the industrial area compared to the other areas, and the functional areas dominated by different land-use types contributed more to Cu accumulation. Additionally, the main risk of the industrial region was mainly caused by Cr, which is affected by human activities such as traffic, mining, and agricultural activities. The results of factor analysis showed that there were four main sources of soil heavy metals in different functional areas. The first was due to human daily activities; the second was soil parent material, the third was industrial production activities, and the fourth was caused by agricultural activities. According to multivariate statistical analysis, Zn, Cu, Hg, and Cr pollution are mainly from mixed sources such as domestic waste and mining activities, Pb is mainly from transportation, and As pollution is mainly from farmland fertilization. These research results can serve as a reference for agricultural environmental protection during urban development.

## Figures and Tables

**Table 1:** Classification criteria for the degree of the single-factor pollution index.

Ranges of *P_i_*	1	2	3	4	5
Pollution level	*P_i_* ≤ 1	1 < *P_i_* ≤ 2	2 < *P_i_* ≤ 3	3 < *P_i_* ≤ 5	*P_i_* > 5
level	Clean	Potential pollution	Mild pollution	Moderate pollution	Heavy pollution

**Table 2:** Classification criteria for the degree of soil heavy metals.

$\mathrm{Ranges}\;\mathrm{of}\;E_{r}^{i}$	<40	40–80	80–160	160–320	>320
Ranges of *PRI*	<90	90–180	180–360	360–720	>720
Ecological risk levels	Low risk	Moderate risk	Considerable risk	High risk	Very high risk

**Table 3:** Statistical description of heavy metal concentrations (mg·kg^−1^) on the northern slope of the Tianshan Mountains in different functional areas.

Area	Elements	Ranges	Mean Value± SD ^a^	CV ^b^	Skewness	Kurtosis	Background Values ^c^	Standard Level ^d^
		(mg·kg^−1^)	(mg·kg^−1^)	(%)			(mg·kg^−1^)	(mg·kg^−1^)
Area A	Zn	43.44~132.06	56.97 ± 16.87	29.61	3.59	15.28	68.8	300
	Cu	17.44~29.44	23.27 ± 3.21	13.79	0.08	−1.06	26.7	100
	Cr	23.81~43.00	33.33 ± 5.30	15.91	0.18	−0.86	49.3	250
	Pb	69.38~129.38	107.87 ± 18.05	16.73	−0.99	−0.45	19.4	170
	As	75.38~126.13	106.91 ± 11.88	11.11	−1.28	2.14	11.2	20
	Hg	0.02~0.05	0.03 ± 0.01	23.92	1.41	2.14	0.02	1.0
Area B	Zn	34.31~73.88	58.00 ± 8.41	14.50	−0.63	0.63	68.8	300
	Cu	16.81~119.19	30.82 ± 16.35	53.05	4.67	25.05	26.7	100
	Cr	23.69~64.94	39.56 ± 9.57	24.19	0.84	0.82	49.3	250
	Pb	45.63~102.50	70.98 ± 10.76	15.16	0.15	1.48	19.4	170
	As	66.88~109.69	89.93 ± 11.74	13.05	−0.06	−0.70	11.2	20
	Hg	0.02~0.05	0.03 ± 0.01	19.38	0.21	−0.78	0.02	1.0
Area C	Zn	20.63~70.38	55.80 ± 7.84	14.04	−1.83	8.51	68.8	300
	Cu	6.38~47.56	28.34 ± 8.67	30.59	−0.45	0.34	26.7	100
	Cr	9.06~66.19	37.80 ± 14.12	37.35	−0.23	−0.50	49.3	250
	Pb	36.25~110.63	81.19 ± 11.98	14.76	−0.97	3.73	19.4	170
	As	6.19~141.63	73.09 ± 27.11	37.10	0.45	0.65	11.2	20
	Hg	0.01~0.06	0.03 ± 0.01	37.95	−0.17	−0.01	0.02	1.0

SD ^a^: standard deviation; CV ^b^: coefficient of variation; regional value ^c^: soil background values of heavy metal in Xinjiang [30]; standard level ^d^: according to the Soil Environmental Quality Management Standard in China (GB15618-2018) [35].

**Table 4:** Correlation coefficients of different functions.

Area	Element	Zn	Cu	Cr	Pb	As	Hg
Area A	Zn	1.00					
	Cu	0.06	1.00				
	Cr	0.10	0.21	1.00			
	Pb	–0.15	–0.49	–0.01	1.00		
	As	–0.002	–0.49	–0.53	0.35	1.00	
	Hg	–0.06	0.45	0.01	–0.46	–0.44	1.00
Area B	Zn	1.00					
	Cu	0.38	1.00				
	Cr	0.45	0.40	1.00			
	Pb	0.09	0.11	0.02	1.00		
	As	–0.58	–0.37	–0.58	–0.01	1.00	
	Hg	0.41	0.35	0.54	0.01	–0.62	1.00
Area C	Zn	1.00					
	Cu	0.66	1.00				
	Cr	0.59	0.80	1.00			
	Pb	0.61	0.32	0.39	1.00		
	As	–0.62	–0.84	–0.85	–0.29	1.00	
	Hg	0.36	0.76	0.79	0.09	–0.80	1.00

**Table 5:** Characteristic value and accumulative contribution of principal component analysis.

Areas	Element	Initial Eigenvalues			Rotated Eigenvalues			After Rotated Component Matrix		
		Total	Variance%	Cumulative%	Total	Variance%	Cumulative%	PC1	PC2	PC3
Area A	Zn	2.45	40.87	40.87	2.45	40.87	40.87	0.11	0.21	0.95
	Cu	1.22	20.26	61.13	1.22	20.26	61.13	0.79	−0.12	0.03
	Cr	1.04	17.36	78.49	1.04	17.36	78.49	0.42	0.84	−0.10
	Pb	0.52	8.72	87.22				−0.70	0.40	−0.27
	As	0.47	7.75	94.97				−0.80	−0.35	0.20
	Hg	0.30	5.04	100				0.70	−0.42	−0.15
Area B	Zn	2.90	48.27	48.27	2.90	48.27	48.27	0.74	0.09	
	Cu	1.03	17.09	65.36	1.03	17.09	65.36	0.63	0.21	
	Cr	0.69	11.54	76.91				0.79	−0.09	
	Pb	0.60	10.01	86.92				0.09	0.97	
	As	0.46	7.63	94.54				−0.85	0.12	
	Hg	0.33	5.46	100.00				0.78	−0.14	
Area C	Zn	4.04	67.38	67.38	4.04	67.38	67.38	0.76	0.48	
	Cu	1.15	19.12	86.51	1.15	19.12	86.51	0.92	−0.12	
	Cr	0.37	6.11	92.61				0.92	−0.11	
	Pb	0.17	2.84	95.45				0.48	0.80	
	As	0.15	2.46	97.91				−0.93	0.18	
	Hg	0.13	2.09	100.00				0.82	−0.46	

## Data Availability

Data are contained within the article.
